# A New Versatile Platform for Assessment of Improved Cardiac Performance in Human-Engineered Heart Tissues

**DOI:** 10.3390/jpm12020214

**Published:** 2022-02-04

**Authors:** Marcelo C. Ribeiro, José M. Rivera-Arbeláez, Carla Cofiño-Fabres, Verena Schwach, Rolf H. Slaats, Simone A. ten Den, Kim Vermeul, Albert van den Berg, José M Pérez-Pomares, Loes I. Segerink, Juan A. Guadix, Robert Passier

**Affiliations:** 1 Department of Applied Stem Cell Technologies, TechMed Centre, University of Twente, 7500 AE Enschede, The Netherlands; m.ribeiro@utwente.nl (M.C.R.); j.m.riveraarbelaez@utwente.nl (J.M.R.-A.); c.cofinofabres@utwente.nl (C.C.-F.); v.schwach@utwente.nl (V.S.); r.h.slaats@utwente.nl (R.H.S.); s.a.tenden@utwente.nl (S.A.t.D.); k.vermeul@utwente.nl (K.V.); 2 River BioMedics, 7522 NB Enschede, The Netherlands; 3 BIOS Lab-on-a-Chip Group, MESA+ Institute for Nanotechnology, Max Planck Institute for Complex Fluid Dynamics, University of Twente, 7500 AE Enschede, The Netherlands; a.vandenberg@utwente.nl (A.v.d.B.); l.i.segerink@utwente.nl (L.I.S.); 4 Department of Animal Biology, Institute of Biomedicine of Málaga (IBIMA), Faculty of Sciences, University of Málaga, 29071 Malaga, Spain; jmperezp@uma.es (J.M.P.-P.); jaguadix@uma.es (J.A.G.); 5 Andalusian Centre for Nanomedicine and Biotechnology (BIONAND), 29071 Malaga, Spain; 6 Department of Anatomy and Embryology, Leiden University Medical Centre, 2300 RC Leiden, The Netherlands

**Keywords:** versatile platform, engineered heart tissues, serum-free, contractile force, cardiac performance, hPSC-CMs

## Abstract

Cardiomyocytes derived from human pluripotent stem cells (hPSC-CMs) hold a great potential as human in vitro models for studying heart disease and for drug safety screening. Nevertheless, their associated immaturity relative to the adult myocardium limits their utility in cardiac research. In this study, we describe the development of a platform for generating three-dimensional engineered heart tissues (EHTs) from hPSC-CMs for the measurement of force while under mechanical and electrical stimulation. The modular and versatile EHT platform presented here allows for the formation of three tissues per well in a 12-well plate format, resulting in 36 tissues per plate. We compared the functional performance of EHTs and their histology in three different media and demonstrated that tissues cultured and maintained in maturation medium, containing triiodothyronine (T3), dexamethasone, and insulin-like growth factor-1 (TDI), resulted in a higher force of contraction, sarcomeric organization and alignment, and a higher and lower inotropic response to isoproterenol and nifedipine, respectively. Moreover, in this study, we highlight the importance of integrating a serum-free maturation medium in the EHT platform, making it a suitable tool for cardiovascular research, disease modeling, and preclinical drug testing.

## 1. Introduction

Cardiovascular disease (CVD) is the leading cause of death globally and its prevalence is expected to continue to rise with the increase of life expectancy [1]. Although large efforts have been made in developing new drugs for the treatment of CVD, translation from basic research to the release of new compounds on the market has diminished greatly in the past two decades due to high clinical trial failure rates [2]. Results from animal models could not be reliably extrapolated to the (patho-)physiology of the human heart and in vitro models using primary cardiomyocytes (CMs) or cell lines do not successfully recapitulate cardiac disease in humans, which underlines the urgent need to create innovative human-based cardiovascular models in order to improve the success rate of discovering new therapeutic approaches. Recent advances in the efficient differentiation of human pluripotent stem cell-derived cardiomyocytes (hPSC-CMs) and other key cardiac cell-types facilitated their application in the development of complex multicellular human pre-clinical cardiovascular models, with a higher resemblance of the human heart and the patient’s disease phenotype [3,4,5].

However, hPSC-CMs are currently limited by their relatively immature phenotype in comparison to human adult CMs. Different studies have shown several approaches to enhance hPSC-CMs maturation in vitro based on key physiological properties from in vivo cardiac development, including the modulation of substrate stiffness; electrical, mechanical, and biochemical stimulation; and three-dimensional (3D) culturing or cell–cell interactions with additional cardiac cell types [6,7,8].

Over the last decades, 3D engineered heart tissues (EHTs) from hPSC-CMs have become a promising and highly advanced model for studying cardiac disease since EHT-CMs exhibit a higher degree of maturation based on various features, including a more defined cellular organization (e.g., sarcomeric assembly and mitochondrial maturation), an expression pattern of maturation-related genes, and an enhanced contractile function, when compared to two-dimensional (2D)-CMs [9,10,11].

However, most EHT protocols rely on undefined culture medium containing serum to generate stable tissues and ensure survival during CM maturation [12,13]. Despite the presence of numerous beneficial components in serum, its undefined composition, batch-to-batch variability, and possible interference with other factors and compounds compromise the standardized formation of defined cardiac tissues and reliable testing [14]. Moreover, the well-known CM hypertrophy-inducing effect of serum can alter the interpretation of experiments investigating disease phenotype or drug responses [15]. Therefore, control over medium composition with defined formulations that support CM maturation might promote optimal cardiomyocyte function and robust readouts. In this regard, we have previously shown that stimulation with biochemical factors with putative roles in CM function, such as the combination of the triiodothyronine hormone (T_3_), dexamethasone (D), and insulin-like growth factor 1 (IGF-1; TDI), in a refined maturation culture medium improves maturation in monolayer cultures with hPSC-CMs [16]. We hypothesize that using these factors in hPSC-derived cardiomyocytes in a 3D configuration, will induce an increase in contractile properties and responses to inotropic agents.

In this study, we developed a versatile platform for the generation and functional analysis of 3D EHTs using hPSC-CMs, suitable for inverted microscopy and requiring a lower number of cells when compared to previously reported conventional EHT platforms [17,18]. We evaluated the formation, organization, and functional performance of EHTs conditioned in various media, including serum-free fully defined maturation medium containing TDI. We demonstrate the importance of biochemical stimulation in enhancing functional properties and the structural morphology of hPSC-CMs in vitro, and establish a serum-free platform which enables standardized drug screening and modeling of cardiac disease in 3D human cardiac tissues (Figure S1).

## 2. Materials and Methods

### 2.1. HPSC Culture and Generation of hPSC-CMs

**Figure 1:**
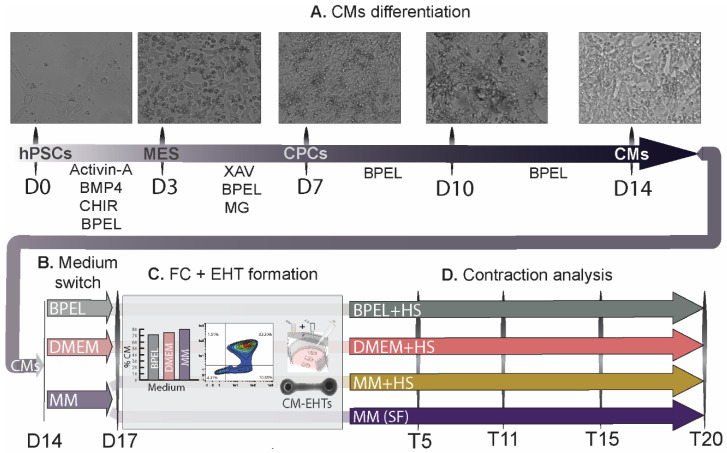
Representative differentiation to CMs from hPSCs and experimental flow chart. (**A**) CM differentiation steps at day 0 (D0), 3 (D3), 7 (D7), 10 (D10), and 14 (D14). (**B**) Medium switch on fully differentiated CMs at day 14 to BPEL, DMEM, or Maturation Medium (MM) until day 17 (D17). (**C**) At day 17, flow cytometry (FC) was performed in CMs from the three different groups, followed by tissue formation in the corresponding media with or without horse serum (HS and SF, respectively). (**D**) Following contraction analysis carried at day 5, 11, 15, and 20 after tissue formation (T5, T11, T15, and T20). Drug tests were performed at the best timepoint of force of contraction assessed (day 10 (D10)). After 21 days of tissue formation (T21), tissues were kept for histological analysis. HPSCs, human pluripotent stem cells; MES, mesoderm; CPCs, cardiac progenitor cells; CMs, cardiomyocytes; MG, Matrigel; HS, horse serum; and SF, serum-free.

This study was performed using a human-induced pluripotent stem cell (hiPSC; LUMC0020iCTRL-06) line and the previously generated double-reporter human embryonic stem cell (hESC) line (HES3) carrying both the Green Fluorescent Protein (GFP) at the NKX2.5 genomic locus [19] and mRubyII fused to the cardiac sarcomeric protein α-actinin (ACTN2; DRRAGN [20]). Undifferentiated hPSCs were maintained in Essential 8 (E8) medium (Thermo Fisher, Lelystad, The Netherlands, A1517001) on vitronectin (Thermo Fisher, A31804)-coated 6-well plates. Cardiac differentiation was induced as described previously [16]. Briefly, on day 1, hPSCs were seeded at a density of 15–25 × 10^3^ cells per cm^2^ on Matrigel (83 µg protein/mL; Corning, Tewksbury, MA, USA, 354230)-coated 6-well plates in E8 medium. After 24 h (day 0 (D0)), mesodermal differentiation was induced by addition of Activin-A (20 ng/mL, Miltenyi, Leiden, The Netherlands, 130–115-010), BMP4 (20 ng/mL, R&D systems, Minneapolis, MN, USA, 314-BP/CF), and Wnt activator CHIR99021 (1.5 μmol/L, Axon Medchem, Groningen, The Netherlands, 1386) in BPEL medium [21]. At day 3 (D3), cells were refreshed with BPEL containing WNT inhibitor XAV939 (5 μmol/L, R&D Systems 3748) and Matrigel (41.3 µg protein/mL). Cells were refreshed with BPEL on day 7 (D7) and 10 (D10) of the differentiation (Figure 1A).

### 2.2. Fabrication of Engineered Heart Tissues (EHT) Platform

**Figure 2:**
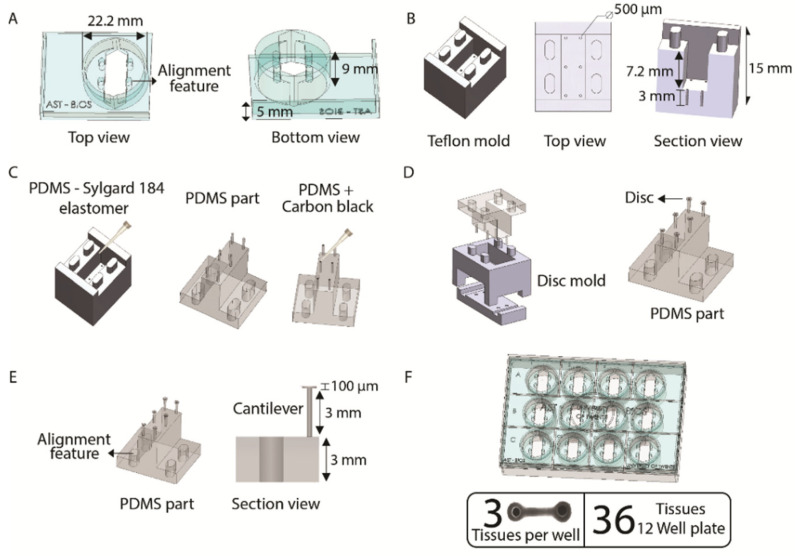
Fabrication of the platform. (**A**) Dimensions of a holder of PMMA in a top and bottom view. (**B**) Schematic of negative Teflon mold of PDMS-part. (**C**) PDMS-part fabrication process. (**D**) Schematic of disc fabrication. (**E**) Illustration of the PDMS mold and dimension of the cantilever. (**F**) EHT platform on a 12-well plate.

The EHT platform has 12 holders and each one of them are made of poly(methyl methacrylate; PMMA), designed in SolidWorks 2018 and fabricated by the Computer Numerical Control (CNC) micro-milling machine (Datron Neo, Mühltal, Germany; Figure 2A). The milling process was performed in two phases: first, the top part of the holder was engraved and milled, which was followed by milling of the bottom part to fit in a well of a CELLSTAR^®^ 12-well cell culture multiwell plate (GreinerBioOne, Alphen aan den Rijn, The Netherlands, cat no. 665180). The depth of each holder was made considering a working volume of 2 mL per well (Figure S2). Each holder supported one polydimethylsiloxane (PDMS) piece containing 6 cantilevers. 

These PDMS pieces with three parallel pairs of cantilevers were made by mold-casting Sylgard 184 (1:10 *v*/*v* ratio, Sigma-Aldrich, St. Louis, MO, USA) into custom-made negative Teflon molds that were designed and fabricated by the CNC micro-milling machine (Figure 2B). Alignment features were included in the design to facilitate a proper fit into the PMMA holders. For the fabrication, the PDMS was casted into the Teflon mold and placed in a vacuum chamber to eliminate bubbles for 45 min and subsequently incubated at 60 °C inside an oven to cure the PDMS overnight. In order to optically track the position of the cantilevers, a drop of the mixture of PDMS with 13% (*w/w*) black carbon (Vulcan XC 72R) was used to stain the top of each one of the six transparent cantilevers. Immediately after, the whole PDMS-part was placed back into the oven at 60 °C for 2 h to cure (Figure 2C). To prevent the EHTs from sliding off the pillars when suspended upside-down, a disc of transparent PDMS of 1 mm of the diameter was made on the end of the cantilever using a custom-made mold from PMMA and cured inside an oven at 60 °C for 2 h (Figure 2D). Each cantilever measures 3 mm in length with a 100 µm thick disc on top connected to a PDMS base of 3 mm (Figure 2E).

With this platform, a total of 36 EHTs were made on a 12-well plate format using 12 holders and 12 PDMS-parts, which means 3 EHTs per well (Figure 2F and Figure S3).

### 2.3. Conditioning of CMs and EHT Formation

At day 14 of differentiation (D14), contracting CMs in monolayers were conditioned with either (1) DMEM culture medium (consisting of DMEM (Biochrom/Sigma-Aldrich, Cambridge, UK, F0415) supplemented with 1% penicillin/streptomycin (Gibco, Lelystad, The Netherlands, 15070063) and 10 µg/mL insulin (Sigma-Aldrich, I9278)), (2) maturation medium (MM; composed of DMEM (Sigma, St. Louis, MO, USA, D5030), 15 mM glucose, 0.5 mM sodium pyruvate, 0.19 mM sodium hydroxybutyrate, 0.5 mM L-carnitine, 1 mM creatine, 5 mM taurine, phenol red (0.011 g/L), 1X Trace elements (A, B, and C; Corning), 1X chemically defined lipids (Life Technologies, Waltham, MA, USA), 2 mM Glutamax, 400 µM α-thioglycerol, 0.1X ITS-X, 50 µg/mL AA-2P, 0.5% Pen-Strep, 3.5 g/L sodium bicarbonate, 100 nM T3, 100 ng/mL Long R3 IGF-1, and 1 µM dexamethasone, as described in [16]) or (3) control medium (BPEL; Figure 1B). After 3 days (day 17 (D17)), cardiac monolayers were dissociated with TrypLE 10X (ThermoFisher, A1217702) for 10 min. CM populations were quantified with flow cytometry (FC) either for cTnT^+^ cells (for hiPSC-CMs) or GFP^+^/mRubyII^+^ (for hESC-CMs). CM differentiation efficiencies from 60% or higher were considered for tissue formation (Figure 1C). 

**Figure 3:**
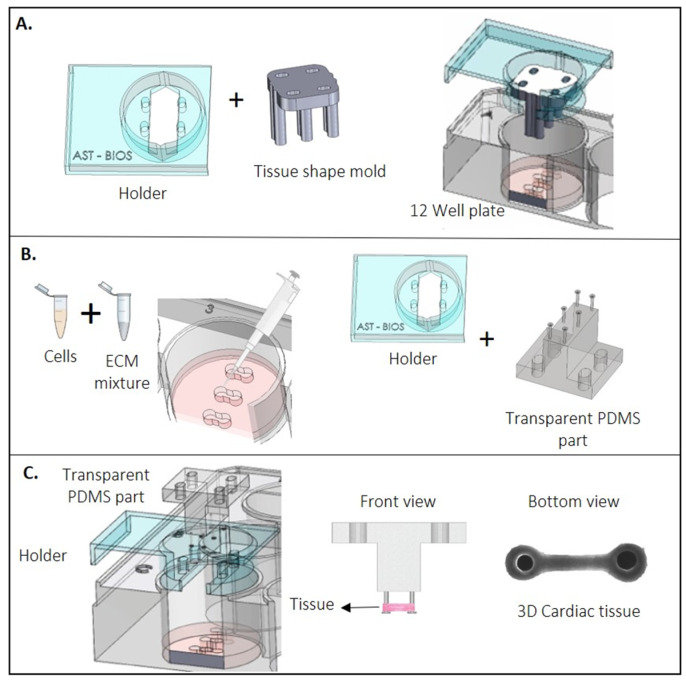
Process of making the EHTs. (**A**) Strip format using a Teflon spacer into a well. (**B**) Diagram of adding the cell suspension with a fibrinogen-based ECM into each tissue slot of a well. (**C**) Schematic of the EHT fabrication.

The EHT formation protocol was adapted from Breckwoldt et al. [22]. A set of 3 tissues (one well of the 12-well plate) was made from CMs of each culture condition by counting and resuspending to a final concentration of 23.6 × 10^6^ cells/mL in the corresponding medium with 10% horse serum (HS; Thermofisher, 26050088) or without (serum-free, SF). Next, an extracellular matrix (ECM) mixture consisting of 2X medium (DMEM, MM, or BPEL), fibrinogen (final concentration of 2 mg/mL, Sigma-Aldrich F8630), Matrigel (final concentration of 1 mg/mL), and aprotinin (final concentration of 2.5 µg/mL, Sigma-Aldrich, A1153) was reconstituted on ice and added to the resuspended cells to get a final cell concentration of 16.3 × 10^6^ cells/mL. Next, 0.6 U/mL of thrombin (Sigma, T7513) was added to the cells + ECM mixture. Quickly after mixing, 15 µL of the final mix (2.45 × 10^5^ cells) was added to the tissue slots. The tissue slots were made using Teflon tissue-shaped molds that were designed and fabricated by a CNC micro-milling machine and were supplemented with 1 mL of the mix of 2X of the specific cell culture condition as well as with 20% (*w*/*v*) of gelatine from porcine skin (G1890-1006) per well. The well plate was placed at 4 °C for 4 h prior to the experiment. Subsequently, the assembled Teflon tissue-shaped molds were removed from the holder (Figure 3A,B). Next, the PDMS part, already assembled in the holder, was placed in the well and the constructs were left at room temperature (RT) for 10 min for fibrinogen polymerization (Figure 3C). Next, 1 mL of the corresponding cell culture medium was added to each well. The EHTs were then maintained at 37 °C and 5% CO_2_ in a humidified cell culture incubator and refreshed the next day. Refreshments were done every 2 or 3 days and after contraction measurements (Figure 1D).

### 2.4. Functional Analysis

To assess compaction of the tissues over time, the surface area of the whole tissue was traced using ImageJ. Next, early-stage compaction was determined by calculating the area of each tissue after 5 days of tissue formation (T5) as a fraction of the seeding surface area at day 0 of tissue formation (T0; 11 mm^2^). Similarly, late-stage compaction was determined by calculating the fractional surface area of each tissue after day 11, 15, and 20 of tissue formation (T11, T15, and T20, respectively) normalized to the T5 area of the same tissue. EHT formation was considered successful when cardiac tissues were homogeneously distributed around the cantilevers without tissue rupture at any timepoint. The success rate was thus defined by the percentage of successful tissues out of the total number of tissues produced.

Force of contraction was measured on day 5, 11, 15, and 20 after tissue formation for a period of 10 s per tissue. Responses to drugs were assessed at day 10. For a positive inotropic response, isoproterenol (Sigma, I5627) was administrated at 3 nM and force of contraction was measured after 5 min of incubation. Gain of force and velocity were assessed with respect to the basal condition (0 nM). Similarly, negative inotropic effects were determined with increasing concentrations of nifedipine (Sigma, N7634; 0–100 µM). After 5 min of each dose’s administration, the tissues were recorded. 

For all measurements, two platinum electrodes (Advent Research Materials) connected to a custom-made pacing device were placed perpendicular to the tissues, approximately 20 mm apart inside of the well. The EHTs were electrically paced at 1 Hz (10 ms biphasic pulses, 4–5 V/cm) while being maintained at 37 °C and 5% CO_2_. Image acquisition was done at 100 fps with 2× magnification. To assess the functional parameters, cantilever deflection in the EHTs was calculated by a custom MatLab-based (version 2020) analysis software developed by our group. To calculate the deflection of the cantilevers over time, the software measured the distance between the black cantilever tops in each frame and compared it with the known initial distance. The contraction force of the EHTs was assessed using the elastic beam bending equation [23].

$$F=\frac{3{\pi \mathrm{ER}}^{4}}{2a^{2}\left(3L-a\right)}\delta$$

where F is the contraction force of the EHT; E, R, and *L* represent Young’s modulus, the radius, and the length of the PDMS cantilever; *a* is the height of the tissue on the cantilever from the base; and δ is the measured distance between cantilevers.

### 2.5. Immunostaining, Histology, and Imaging

At day 21, EHTs cultured with different treatments were fixed in 4% paraformaldehyde (PFA) in phosphate-buffered saline (PBS) for 30 min at RT for the sectioning procedure or 1 h for whole-mount staining. 

For whole-mount staining, EHTs after fixation were washed with 0.3% Triton-X 100 (Sigma-Aldrich; 3 × 20 min), blocked for non-specific binding with 3% BSA, 0.3% Triton-X 100, and 0.1% Tween in PBS overnight at 4 °C. Primary antibody anti-cardiac troponin T (1:400; Invitrogen, MA5-12960), anti-connexin-43 (1:200; Sigma-Aldrich, C6219), or anti-cardiac troponin I3 (1:800; Abcam, ab10231) were then incubated for 2 days at 4 °C. Then, tissues were washed with 0.3% Triton-X 100 (3 × 20 min) and secondary antibody Goat-anti-Mouse IgG Alexa Fluor 647 (1:500; Invitrogen, A21235), and/or Goat-anti Rabbit IgG Alexa Fluor 488 (1:500; Invitrogen, A27034) and DAPI were added for 24 h at 4 °C. After three washes with PBS, tissues were mounted on a microscope slide for confocal imaging with a Zeiss LSM 880 microscope. 

For cryo and paraffin sections, after fixation, EHTs were extensively washed in PBS and either cryoprotected by overnight incubation in 30% sucrose; embedded in CryoMatrix Gel (Thermo Scientific, 6769006) and 12 µm sectioned in an MNT (SLEE Medical) cryostat; or dehydrated in ethanol, cleared in butanol, embedded in Histosec (Merk), and 10 µm sectioned in a Leica microtome. Immunohistochemical characterization of CMs in cryosections was performed using a Mouse monoclonal anti-alpha-actinin (ACTN2) antibody (Sigma, A7811). Tissue sections were permeabilized with PBS containing 0.1% Triton-X 100 (Sigma-Aldrich) for 8 min and blocked for 1 h with PBS containing both 1% (*vol/vol*) BSA and 5% (*vol/vol*) goat serum. Sections were incubated with the primary antibody α-actinin (1:800) overnight at 4 °C. Then, the samples were washed in PBS solution (3 × 5 min) and incubated with the secondary antibody Goat-anti-Mouse IgG Alexa Fluor 647 (Invitrogen, A21235) or Goat-anti-Mouse IgG Alexa Fluor 488 (Invitrogen, A11001) for 1 h at room temperature. After three washing steps with PBS (20 min each), tissues were counterstained with DAPI (4′,6-diamidine-2-phenylidole-dihydrochloride; Thermo Scientific). Finally, the slides were mounted with ProLong Gold antifade with DAPI (Life Technologies) and analyzed under a Zeiss LSM 880 laser confocal microscope.

Immunostaining on paraffin sections was carried out by blocking non-specific binding sites with SBT (TPBS1X, Goat Serum, Albumin, and Triton X-100) and incubating the slides in the primary cardiac troponin-I (cTnT1) antibody (1/100 diluted in SBT, Santa Cruz sc-133117) overnight at 4 °C. Then, the slides were washed in PBS solution (3 × 5 min) and incubated for 2 h at RT in Cy5 AffiniPure Donkey-anti-Mouse immunoglobulin G (IgG; 1/200 diluted in PBS, Jackson 715-175-150). Nuclear DAPI counterstaining was performed. Finally, the slides were mounted and analyzed under a LEICA SP5 laser confocal microscope.

### 2.6. Gene Expression 

For RT-qPCR, RNA from hESC-EHTs in each media at day 21 was purified using the Nucleo Spin RNA (Macherey-Nagel) according to the manufacturer’s protocol and reverse-transcribed to cDNA using the iScript cDNA Synthesis kit (Bio-Rad). Gene expression was assessed using a Bio-Rad CFX384 real time system using SensiMix SYBR (Meridian). The samples were normalized to the house keeping gene human RPLP0. Primer sequences used can be found in Supplementary Table S1. 

### 2.7. Statistics

Statistical analysis was performed using GraphPad Prism 8. Each experiment was performed 4 times, with CMs from 4 independent differentiations. Per experiment, each set of 3 tissues (one well of a 12wp) was considered as technical replicates. 

Differences between groups were assessed by two-way ANOVA plus Tukey’s post-hoc test and for comparison within one medium by one-way ANOVA plus Tukey’s post-hoc test. Results are displayed as mean ± SEM unless stated otherwise. Significance was attributed to comparisons with values of *p* < 0.05 ¥; *p* < 0.01 †; *p* < 0.001 ‡; and *p* < 0.0001 #.

## 3. Results

### 3.1. Fabrication of the Platform

The EHT platform consisting of 12 individual PMMA-based holders was optimized to fit perfectly into a standard 12-well culture plate (Figure 2F). Each holder contains four alignment features (Figure 2A) that allowed for the successful assembling and aligning of both the tissue mold (Figure 3A) and the PDMS-part into the holder. As a result, the six cantilevers on the PDMS-part can settle into the tissue slot, enabling the proper formation of the EHTs around them (Figure 3C). The disc on top of the cantilevers combined with the black marks successfully supported the EHTs and enabled tracking of the position of the cantilevers during repetitive cycles of contraction (Figure S3).

### 3.2. Maturation Medium Improves Tissue Formation and Induces an Increase in Contraction Force

**Figure 4:**
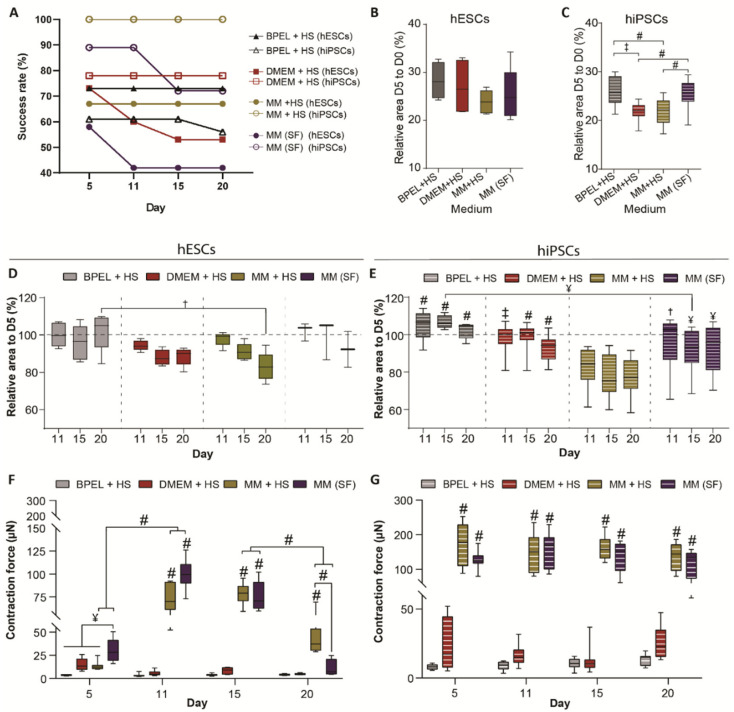
EHT comparison in different media with and without HS. (**A**) Success rate of tissue formation in hESCs and hiPSCs. (**B**,**C**) Early-stage compaction measured by comparing the tissue area at day 5 with the initial area at day 0 in each condition in hESCs (**B**) and hiPSCs (**C**). (**D**,**E**) Late-stage compaction measured by comparing the tissue area at day 11, 15, and 20 with its area at day 5 in the different media in hESCs (**D**) and hiPSCs (**E**). In (**E**), *p*-values indicate significant differences compared with MM+HS within the same timepoint unless otherwise indicated. (**F**,**G**) Contractile force of EHTs in each medium in hESCs (**F**) and hiPSCs (**G**) at day 5, 11, 15, and 20. In (**F**,**G**), *p*-values represented without significance line indicate significant differences versus BPEL+HS and DMEM+HS. HESCs data are shown as means, maxima, and minima. Two-way ANOVA plus Tukey’s test for comparisons among media and one-way ANOVA plus Tukey’s test for comparisons within one medium: ¥ = *p* < 0.05; † = *p* < 0.01; ‡ = *p* < 0.001; and # = *p* < 0.0001 (*N* = 4, biological replicates from independent differentiations). HS, horse serum and SF, serum free.

In order to improve the current contractile output of EHTs, we made use of our previously developed defined maturation medium (MM) containing T_3_, IGF-1, and dexamethasone [16], and compared it to the standard EHT medium (DMEM) [17] and to our CM differentiation medium (BPEL). Since the standard EHT medium includes horse serum, we supplemented the tissues in MM and BPEL media with 10% horse serum (+HS) as well. To assess the variation of the tissue formation and functional analysis under these different culture conditions across different hPSC lines, we used both hiPSC (LUMC0020iCTRL-06) and hESC (HES3 NKX2.5^eGFP/w^) lines. Prior to tissue formation, CM differentiation was evaluated after three days (D17) of conditioning the CM monolayers in the corresponding media without HS (Figure 1B,C). While the CMs of each medium condition originated from the same batch of differentiation, the average percentage of CMs in the population varied substantially between media after the conditioning step. Consequently, EHTs from hiPSC-CMs were formed with a percentage of CMs of 68 ± 6%, 79 ± 13%, and 85 ± 7% in BPEL, DMEM, or MM, respectively (Figure S4A,B). Similarly, EHTs were made of hESC-CMs with percentages of 73 ± 9%, 76 ± 6%, and 81 ± 7% in BPEL, DMEM, or MM, respectively (Figure S4C,D). Once the tissues were made, the formation of the EHTs from hESC-CMs and hiPSC-CMs over time showed that the first 5 days of cell culture are vital for the success rate of tissue in formation over the next 20 days. The highest success rate for EHT formation was achieved with hiPSCs in MM+HS (100%, 16 of 16 tissues), followed by DMEM+HS with 80% (14 of 16 tissues) and BPEL+HS with only 56% (10 of 16 tissues). Using hESC-CMs, the success formation of EHTs was, on average, lower than hiPSC-CMs, although the highest success rate was found in BPEL+HS with 73% (11 of 15 tissues), closely followed by MM+HS with 67% (10 of 15 tissues) and DMEM+HS with 53% (8 of 15 tissues; Figure 4A).

It has been shown that for optimal contractile output, remodeling and compaction of tissue plays an important role [24]. In order to evaluate tissue remodeling during culture, we next analyzed how the EHTs compacted over time by measuring the tissue area at each timepoint. Since compaction is mostly determined within the first 5 days, analysis was divided in two parts: the first stage of the compaction of tissues from day 0 to 5 (early-stage compaction) and the second stage of additional relative compaction from day 5 onwards (late-stage compaction). Both hESC-EHTs and hiPSC-EHTs compacted mostly in MM+HS, reducing the surface area by 70–75% during early-stage compaction and making hiPSC-EHTs significantly smaller than in other medium types (Figure 4B,C). Late-stage tissue compaction was also most effective in MM+HS compared to BPEL+HS and DMEM+HS conditions. It is worthy to note that hESC-EHTs showed the highest compaction in MM+HS at day 20 (Figure 4D), whereas hiPSC-EHTs already reached maximum compaction at day 11 (Figure 4E).

There is a general interest and preference to perform experiments in serum-free cultures in order to avoid possible interference of serum-borne factors with drugs or other agents and the interference or induction of disease-related mechanisms (such as cardiac hypertrophy [15]). Based on our findings that EHTs cultured in MM+HS yielded the highest success rate in EHT formation and displayed the best functional performance (see below), we decided to evaluate whether EHT formation and functional aspects were maintained in a completely defined serum-free MM (MM(SF)). Accordingly, at day 5, hiPSC-EHTs formed with a success rate of 90% in MM(SF); after day 11, the success decreased to 70% (13 of 16 tissues). To a lesser extent, 60% of hESC-EHTs were successfully formed until day 5 and thereafter the rate dropped to 40% (5 of 12 tissues; Figure 4A). Early-stage compaction of hESC-EHTs in MM(SF) was comparable to the three media containing serum (Figure 4B). In contrast, hiPSC-EHTs in MM(SF) compacted 5% less at the early stage compared to MM and DMEM with serum (+HS; Figure 4C). Both ESC and hiPSC-EHTs in MM(SF) did not further compact from day 5 onwards (Figure 4D,E). 

Next, contraction of the cardiac tissues was visible at day 3 and first functional parameters were measured at day 5 in the different media. Additionally, these parameters were measured at three other timepoints (on day 11, 15, and 20) to evaluate tissue performance over time. The contractile force of hiPSC-EHTs was comparable between MM+HS and MM(SF), and it was significantly higher than BPEL+HS and DMEM+HS at all timepoints. On the other hand, hESC-EHTs in MM(SF) presented a significantly higher contraction force from day 5 to day 15 compared to BPEL+HS and DMEM+HS, while in MM+HS, this significant increase was observed from day 11 to day 20 (Figure 4F,G). 

At every timepoint, the contraction force was higher in hiPSC-EHTs in MM+HS and MM(SF) than in hESC-EHTs in all the media, except for MM(SF) at day 11.

Besides contraction force, the velocity of contraction and relaxation are important parameters for assessing CM maturation [6,16,25,26] and were analyzed in both hESC and hiPSC-EHTs. Both hESC and hiPSC-EHTs displayed significantly higher contraction and relaxation velocities at different times in MM+HS and MM(SF) media when compared to other media, with more consistent and prominent effects at all timepoints in the case of hiPSC-EHTs (Figure S5). Accordingly, 10% and 90% of contraction times were lower in MM+HS and MM(SF) with both hESC and hiPSC-EHTs (Figure S6A–D). In terms of relaxation times, MM(SF) on hESC and hiPSC-EHTs showed lower times to achieve 10% and 90% of relaxation compared to MM+HS (Figure S6E–H).

### 3.3. EHTs in Maturation Medium Respond to Positive and Negative Inotropic Agents

**Figure 5:**
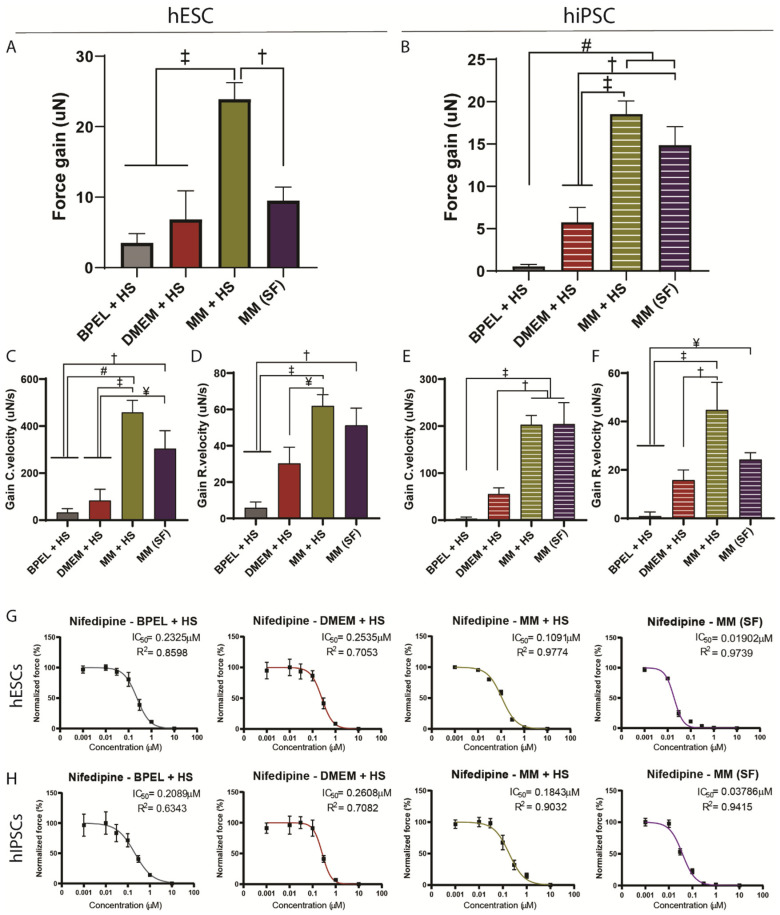
Response to inotropic agents in hESC and hiPSC-EHTs. (**A**–**D**) Absolute gain of contraction force of hESCs (**A**) and hiPSCs (**B**). EHTs in different media in response to isoproterenol (3 nM). (**C**–**F**) Absolute gain of contraction (Gain C.velocity) and relaxation (Gain R.velocity) velocity of hESCs (**C**,**D**) and hiPSCs (**E**,**F**). EHTs in different media in response to isoproterenol (3 nM). (**G**,**H**) Normalized force of contraction of hESCs (**G**) and hiPSCs (**H**). EHTs in different media in response to increasing concentrations of nifedipine (0–10 µM). (**A**–**F**) Data shown as one-way ANOVA plus Tukey’s test. Values are expressed as means ± SEM: ¥ = *p* < 0.05; † = *p* < 0.01; ‡ = *p* < 0.001; and # = *p* < 0.0001 (*N* = 4, biological replicates from independent differentiations). HS, horse serum and SF, serum free.

A positive inotropic response to β-adrenergic agonists, such as isoproterenol, is an important readout of maturity in CMs [12,27,28]. Administration of 3nM isoproterenol led to a significant increase of contraction force as well as contraction and relaxation velocities in MM+HS for both hESC and hiPSC-EHTs, and also in MM(SF) for hiPSC-EHTs (Figure 5A–F). In a similar fashion, hESC-EHTs in MM(SF) presented a gain of force with the β-adrenergic agonist but this was not significantly different than BPEL+HS and DMEM+HS (Figure 5A). On the other hand, there was a significant gain of contraction and relaxation velocities compared to those two media (Figure 5C,D). 

To further assess the EHT response to another inotropic agent under the different medium conditions, nifedipine was tested. Accordingly, a blockade of the L-type Ca^2+^ current upon increasing concentrations of nifedipine (0–10 µM) resulted in a dose-dependent reduction of contraction force in all four media in both hESC and hiPSC-EHTs (Figure 5G,H and Figure S7A,B), with a concomitant drop in contraction and relaxation velocities (Figure S7C–F). Notably, MM+HS-cultured EHTs were more sensitive to nifedipine compared to BPEL+HS and DMEM+HS, as showed by a lower half-maximal inhibitory concentration (Figure 5G,H). Most importantly, tissues in MM(SF) presented an even higher sensitivity to nifedipine compared to all other counterparts with five to ten-folds lower IC50.

### 3.4. Maturation Medium Improves Tissue Morphology and Gene Expression

**Figure 6:**
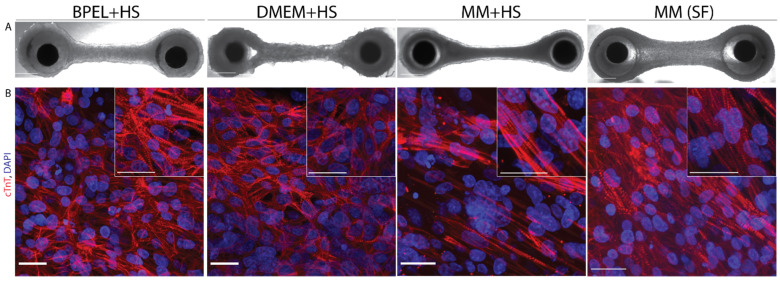
Morphological analysis of EHTs in four different media at day 21 of culture. (**A**) Brightfield images of full EHTs in BPEL+HS, DMEM+HS, MM+HS, or MM(SF). Scale bar = 0.5 mm. (**B**) Confocal images of whole-mount tissue immunostaining for cardiac troponin T (cTnT, red) counterstained with DAPI (nuclei, blue). Scale bars, 25 µm. HS, horse serum and SF, serum-free.

Histological sections of EHTs were analyzed at the central area of the tissues at day 21 (Figure 6A). Immunohistochemical analysis revealed differences in the cardiomyocyte morphology and distribution across the different culture media (Figure 6B). Tissues in both serum-free MM(SF) and MM+HS revealed cardiomyocytes aligned to the longitudinal axis of the tissues and contained better-organized striated sarcomeres. Notably, these organized cardiomyocytes were mostly present on the outer layers of the tissues. In comparison, EHTs in DMEM+HS also revealed cardiomyocytes aligned to the longitudinal axis of the tissues, although without the presence of well-organized sarcomere structures. Finally, EHTs in BPEL+HS revealed a network of randomly organized cardiomyocytes without the presence of well-organized sarcomere structures (Figure 6A,B and Figure S8). Expression of Connexin-43 was also more pronounced in EHTs in MM(HS) and MM(SF) media when compared to BPEL(HS) and DMEM(HS), although the pattern was more variable (Figure S8E). In all cases, no necrotic core was observed in the inner part of the tissues (Figure S8F). 

Next to the functional and morphological observations of the EHTs in different culture conditions, we also analyzed the expression of genes encoding for sarcomeric proteins MYH7, TNNI3, and ACTN2, and for calcium-handling proteins SERCA2 and RYR2. We observed an increased expression of these genes in EHTs cultured in MM+HS. A similar trend was observed in MM(SF) also (Figure S9). Moreover, PGC1α expression levels were also upregulated in both MM+HS and MM(SF). Taken together, these results suggest that the higher functional performance and sarcomere organization observed in both MM+HS and MM(SF) also correlate to changes in the gene expression of the cardiomyocytes that can be attributed to an enhanced state of maturation. 

## 4. Discussion

Advanced human cardiac tissue models hold great promise in disease modeling and drug screening, prospectively leading to new therapeutic approaches and personalized medicine. In order to fulfill this potential, it is imperative to develop assays that allow for robust and reproducible production as well as analysis of these cardiac tissues according to good manufacturing practices [29].

In this study we have established a medium throughput platform for the production and evaluation of 36 EHTs that fit in a standard 12-well plate. This platform has the advantage of 12 modular, independent holders that can be used one at a time or in multiple configurations, which is beneficial for addressing multiple biological questions at the same time in independent wells. Furthermore, in each individual holder, three EHTs can be made, accounting for technical replicates under the same experimental condition. The currently available EHT models or platforms which use a comparable cylindrical flexible cantilever in a similar format produce a lower number of EHTs (1 per custom-made bioreactor and 24 in 24-well plates) [17,18] or do not fit in a commercial culture plate. Instead, we used 245 × 103 cells per EHT, requiring approximately nine million cells for the production of 36 tissues (additional cells are needed in the process of tissue formation). Moreover, for functional analysis, our EHT platform can be easily integrated with a common inverted microscope setup and incubation chamber as opposed to the custom mounted camera setup in the aforementioned conventional EHT platform [17].

We next applied our EHT platform to address two weaknesses of in vitro cardiac 3D models commonly reported by the field. First, for the generation of EHTs and in CM culture in general, fetal bovine serum or HS is often used as a standard additive in media to provide growth factors, nutrients, and hormones in order to improve CM viability and contractile activity [14,29,30]. However, it is also known that serum affects cell growth, differentiation, morphology, and the signaling of CMs, and the batch-to-batch variability of serum may lead to biased outcomes [14]. Moreover, cardiac tissues engineered in vitro in serum containing conditions cannot be readily used in the clinic because of the presence of xenogenic additives. Furthermore, proteins in serum may bind to drugs and significantly affect functional properties in drug-screening platforms [31]. During the formation of EHTs, we observed a significant decrease in the cross-sectional area of the tissue from day 0 to 5. According to previous findings, this initial area reduction is mainly related to fibrin compaction and tissue remodeling, where matrix compaction is reported to occur from day 3 up to one week after tissue formation [11,18,32]. After day 5, only EHTs in serum-containing MM further continued to compact. Although the mechanism responsible for late-stage compaction is currently unknown, growth factors and adhesion factors that are enriched in serum may create more anchoring points and alter the ECM composition of EHTs compared to serum-free conditions [33]. Moreover, serum-containing medium enhances the growth and proliferation of other non-CM cell types [34], which are required to compact into a functional tissue [13,18].

Second, in this study, we addressed the relative immature nature of the hPSC-CMs by culturing the EHTs in a culture medium containing stimulants for maturation (Maturation Medium). Many studies have shown that hPSC-CMs are immature and therefore not directly comparable to adult human CMs. It has been shown that T_3_ and insulin in the absence of serum may play a role in the sarcomeric organization and preventing of CM apoptosis in EHTs, respectively [35]. Accordingly, tissues in MM, both with and without serum, presented a more homogeneous distribution of CMs and a better sarcomere organization when compared to the other tissues, which is a hallmark for cardiomyocyte maturation. 

Next to more organized tissue formation, increased contraction force is an important functional hallmark of cardiac maturation. We previously observed a significant increase in the contraction force of 2D single cell CM cultures from both hiPSC and hESC lines that were maintained in MM [36]. Although in our platform the CMs retained the auto-pacing phenotype, which is a sign of relative immaturity, we observed that the force of contraction levels in hiPSC-EHTs in MM+HS were 30% higher than the reported values using standard EHT medium (DMEM) supplemented with serum [12]. Interestingly, comparable to MM+HS, similar or slightly lower levels of force of contraction were achieved in MM(SF). It is noteworthy that although the contraction forces of hESC-EHTs in the different media were lower than those of hiPSC-EHTs, levels were consistently higher in serum-free and serum-containing MM media. With respect to the lower contractile force in hESC-EHTs, it has been reported that hESC-CMs derived from the same parental hESC line as used in our current study were electrophysiologically less mature than hiPSC-CMs when cultured under the same conditions [37]. These findings indicate that the increase in maturation level through medium composition can be achieved regardless of the starting maturation level of each hPSC line.

Another indicator of CM maturation is the positive inotropic response (increased force) to the β-adrenoceptor agonist isoproterenol [27,38]. HESC and hiPSC-EHTs in MM+HS showed a gain of force in response to isoproterenol. This response was also observed in MM(SF) in hiPSC-EHTs. Accordingly, a gain in the contraction and relaxation velocity was observed in MM+HS and MM(SF). Although these results are indicative of cardiac maturation, these responses are still smaller compared to human heart tissue (difference of 200%) [39], indicating that additional steps of maturation are required. Additionally, EHTs also responded well to negative inotropy induced by the L-type Ca^2+^ channel agonist nifedipine. Sensitivity to nifedipine was higher in both hESC and hiPSC-EHTs in MM compared to the other media and closer to sensitivity values reported in human adult primary cardiomyocytes [40]. Moreover, serum-free MM displayed a clear increase in sensitivity to nifedipine compared to serum-containing MM, which supports the concept that a serum-free platform for drug-screening would be beneficial. 

In agreement with the increased force of contraction, gene expression analysis of the EHTs in MM+HS and MM(SF) revealed an increased expression of genes encoding for the contractile sarcomeric proteins ACTN2, MYH7, and TNNI3, which have been previously related to cardiomyocyte maturation [6]. Moreover, the upregulation of PGC1α in EHTs in these two media suggests an improvement in the cardiac tissue’s bioenergetics since this gene has been identified as a major regulator of mitochondrial function [41]. In short, this study demonstrates that EHTs could be formed with a higher degree of organization and improved contractile performance by applying an improved cell culture medium, omitting undefined serum components but supplementing CM maturation factors. Since EHTs in MM+HS performed best, future improvements of culture medium could be made by evaluating which factors in horse serum contribute to a better performance. The design of the EHT platform enabled us to rapidly screen multiple culture medium conditions, a trait that makes it well-suited to systematically study other maturation factors, such as the effect of multicellularity in various ratios and both combinations and adaptations in energy sources as already described [13,42,43,44], with contractile force as a major functional readout. Improvements in developing robust and mature hiPSC-EHTs will not only lead to a better understanding of the underlying mechanisms of cardiac maturation but will also serve as a platform for studying contractile function in patient-specific diseases, which, in addition to the cardiovascular field, can also be extended to the skeletal muscle field. Furthermore, the medium-throughput level of this platform enables its use for drug screening. With the technology and approach that we have used here to establish a serum-free EHT platform for standardized functional analysis, it will be feasible to up-scale and miniaturize EHTs and address these issues in follow-up studies, which will represent an important step towards predictable disease modelling of cardiovascular disease, safety pharmacology, and drug discovery.

## 5. Conclusions

To conclude, we presented a modular platform for the production of 3D cardiac tissues (EHTs) that fits into a commercial 12-well plate, providing three technical replicates per well, with a total of 36 EHTs per plate. Moreover, this EHT platform easily integrates with a common inverted microscope set up in combination with automatic analysis using optical tracking, which provides force, velocity, and the time of contraction or relaxation as functional readouts. We observed improvement in the formation and functional performance (both contraction and relaxation parameters) of EHTs cultured in maturation medium, even in the absence of serum. This EHT platform will facilitate and expedite the standardization and validation of advanced human cardiac function in vitro, which will be beneficial for cardiac disease modeling and drug discovery.

## Data Availability

Data supporting this article can be provided by authors upon reasonable request.

## Supplementary Materials

The following are available online at https://www.mdpi.com/article/10.3390/jpm12020214/s1, Figure S1: Schematic of the main advantages of the versatile platform for assessment of human engineered heart tissues; Figure S2: Holders fabrication from PMMA by a milling machine; Figure S3: Bioengineered platform; Figure S4: Cardiomyocyte differentiation; Figure S5: Contraction & Relaxation velocity in different media; Figure S6: Contraction & Relaxation time in different media; Figure S7: Response to negative inotropic agents in hESC and hiPSC EHTs; Figure S8: Morphological analysis of EHTs; Figure S9: Relative gene expression of cardiac gen es for hESC CM BPEL+HS, DMEM+HS, MM+HS or MM(SF) at day 21 and Table S1: Primer sequences for RT-qPCR.

## References

[1] Benjamin E.J., Muntner P., Alonso A., Bittencourt M.S., Callaway C.W., Carson A.P., Chamberlain A.M., Chang A.R., Cheng S., Das S.R. (2019). Heart disease and stroke statistics—2019 update: A report from the American heart association. Circulation.

[2] Van Norman G.A. (2017). Overcoming the Declining Trends in Innovation and Investment in Cardiovascular Therapeutics. JACC Basic Transl. Sci..

[3] Lian Q., Chow Y., Esteban M.A., Pei D., Tse H.-F. (2010). Future perspective of induced pluripotent stem cells for diagnosis, drug screening and treatment of human diseases. Thromb. Haemost..

[4] Burridge P.W., Matsa E., Shukla P., Lin Z.C., Churko J.M., Ebert A.D., Lan F., Diecke S., Huber B., Mordwinkin N.M. (2014). Chemically defined generation of human cardiomyocytes. Nat. Methods.

[5] Passier R., Orlova V., Mummery C. (2016). Complex Tissue and Disease Modeling using hiPSCs. Cell Stem Cell.

[6] Yang X., Pabon L., Murry C.E. (2014). Engineering Adolescence. Circ. Res..

[7] Kolanowski T.J., Antos C.L., Guan K. (2017). Making human cardiomyocytes up to date: Derivation, maturation state and perspectives. Int. J. Cardiol..

[8] Ahmed R.E., Anzai T., Chanthra N., Uosaki H. (2020). A Brief Review of Current Maturation Methods for Human Induced Pluripotent Stem Cells-Derived Cardiomyocytes. Front. Cell Dev. Biol..

[9] Hansen A., Eder A., Bönstrup M., Flato M., Mewe M., Schaaf S., Aksehirlioglu B., Schwörer A., Uebeler J., Eschenhagen T. (2010). Development of a Drug Screening Platform Based on Engineered Heart Tissue. Circ. Res..

[10] Eschenhagen T., Eder A., Vollert I., Hansen A. (2012). Physiological aspects of cardiac tissue engineering. Am. J. Physiol. Circ. Physiol..

[11] Nunes S.S., Miklas J., Liu J., Aschar-Sobbi R., Xiao Y., Zhang B., Jiang J., Massé S., Gagliardi M., Hsieh A. (2013). Biowire: A platform for maturation of human pluripotent stem cell–derived cardiomyocytes. Nat. Methods.

[12] Mannhardt I., Breckwoldt K., Letuffe-Brenière D., Schaaf S., Schulz H., Neuber C., Benzin A., Werner T., Eder A., Schulze T. (2016). Human Engineered Heart Tissue: Analysis of Contractile Force. Stem Cell Rep..

[13] Zhao Y., Rafatian N., Feric N.T., Cox B.J., Aschar-Sobbi R., Wang E.Y., Aggarwal P., Zhang B., Conant G., Ronaldson-Bouchard K. (2019). A Platform for Generation of Chamber-Specific Cardiac Tissues and Disease Modeling. Cell.

[14] van der Valk J., Brunner D., De Smet K., Svenningsen Å.F., Honegger P., Knudsen L.E., Lindl T., Noraberg J., Price A., Scarino M.L. (2010). Optimization of chemically defined cell culture media—Replacing fetal bovine serum in mammalian in vitro methods. Toxicol. In Vitro.

[15] Dambrot C., Braam S.R., Tertoolen L.G.J., Birket M., Atsma D.E., Mummery C.L. (2014). Serum supplemented culture medium masks hypertrophic phenotypes in human pluripotent stem cell derived cardiomyocytes. J. Cell. Mol. Med..

[16] Birket M., Ribeiro M.C., Kosmidis G., Ward D., Leitoguinho A.R., van de Pol V., Dambrot C., Devalla H.D., Davis R., Mastroberardino P.G. (2015). Contractile Defect Caused by Mutation in MYBPC3 Revealed under Conditions Optimized for Human PSC-Cardiomyocyte Function. Cell Rep..

[17] Schaaf S., Shibamiya A., Mewe M., Eder A., Stoehr A., Hirt M.N., Rau T., Zimmermann W.-H., Conradi L., Eschenhagen T. (2011). Human Engineered Heart Tissue as a Versatile Tool in Basic Research and Preclinical Toxicology. PLoS ONE.

[18] Cashman T., Josowitz R., Gelb B.D., Li R.A., Dubois N.C., Costa K.D. (2016). Construction of Defined Human Engineered Cardiac Tissues to Study Mechanisms of Cardiac Cell Therapy. J. Vis. Exp..

[19] A Elliott D., Braam S.R., Koutsis K., Ng E.S., Jenny R., Lagerqvist E.L., Biben C., Hatzistavrou T., E Hirst C., Yu Q.C. (2011). NKX2-5eGFP/w hESCs for isolation of human cardiac progenitors and cardiomyocytes. Nat. Methods.

[20] Ribeiro M.C., Slaats R.H., Schwach V., Rivera-Arbelaez J.M., Tertoolen L.G., van Meer B.J., Molenaar R., Mummery C.L., Claessens M.M., Passier R. (2020). A cardiomyocyte show of force: A fluorescent alpha-actinin reporter line sheds light on human cardiomyocyte contractility versus substrate stiffness. J. Mol. Cell. Cardiol..

[21] Ng E.S., Davis R., Stanley E.G., Elefanty A. (2008). A protocol describing the use of a recombinant protein-based, animal product-free medium (APEL) for human embryonic stem cell differentiation as spin embryoid bodies. Nat. Protoc..

[22] Breckwoldt K., Letuffe-Brenière D., Mannhardt I., Schulze T., Ulmer B., Werner T., Benzin A., Klampe B., Reinsch M., Laufer S. (2017). Differentiation of cardiomyocytes and generation of human engineered heart tissue. Nat. Protoc..

[23] Serrao G.W., Turnbull I.C., Ancukiewicz D., Kim D.E., Kao E., Cashman T., Hadri L., Hajjar R.J., Costa K.D. (2012). Myocyte-Depleted Engineered Cardiac Tissues Support Therapeutic Potential of Mesenchymal Stem Cells. Tissue Eng. Part A.

[24] Boudou T., Legant W.R., Mu A., Borochin M.A., Thavandiran N., Radisic M., Zandstra P.W., Epstein J.A., Margulies K.B., Chen C. (2012). A Microfabricated Platform to Measure and Manipulate the Mechanics of Engineered Cardiac Microtissues. Tissue Eng. Part A.

[25] Rodriguez M.L., Graham B.T., Pabon L.M., Han S.J., Murry C.E., Sniadecki N.J. (2014). Measuring the Contractile Forces of Human Induced Pluripotent Stem Cell-Derived Cardiomyocytes With Arrays of Microposts. J. Biomech. Eng..

[26] Hayakawa T., Kunihiro T., Ando T., Kobayashi S., Matsui E., Yada H., Kanda Y., Kurokawa J., Furukawa T. (2014). Image-based evaluation of contraction–relaxation kinetics of human-induced pluripotent stem cell-derived cardiomyocytes: Correlation and complementarity with extracellular electrophysiology. J. Mol. Cell. Cardiol..

[27] Ronaldson-Bouchard K., Ma S.P., Yeager K., Chen T., Song L., Sirabella D., Morikawa K., Teles D., Yazawa M., Vunjak-Novakovic G. (2018). Advanced maturation of human cardiac tissue grown from pluripotent stem cells. Nature.

[28] Sala L., Van Meer B.J., Tertoolen L.G., Bakkers J., Bellin M., Davis R.P., Denning C., Dieben M.A.E., Eschenhagen T., Giacomelli E. (2017). Versatile open software to quantify cardiomyocyte and cardiac muscle contraction in vitro and in vivo. bioRxiv.

[29] Pamies D., Bal-Price A., Chesne C., Coecke S., Dinnyes A., Eskes C., Grillari R., Gstraunthaler G., Hartung T., Jennings P. (2018). Advanced Good Cell Culture Practice for human primary, stem cell-derived and organoid models as well as microphysiological systems. ALTEX.

[30] Eschenhagen T., Didié M., Münzel F., Schubert P., Schneiderbanger K., Zimmermann W.H. (2002). 3D engineered heart tissue for replacement therapy. Basic Res. Cardiol. Suppl..

[31] Schocken D., Stohlman J., Vicente J., Chan D., Patel D., Matta M.K., Patel V., Brock M., Millard D., Ross J. (2018). Comparative analysis of media effects on human induced pluripotent stem cell-derived cardiomyocytes in proarrhythmia risk assessment. J. Pharmacol. Toxicol. Methods.

[32] Goldfracht I., Protze S., Shiti A., Setter N., Gruber A., Shaheen N., Nartiss Y., Keller G., Gepstein L. (2020). Generating ring-shaped engineered heart tissues from ventricular and atrial human pluripotent stem cell-derived cardiomyocytes. Nat. Commun..

[33] Fisher J.P., Mikos A.G., Bronzino J.D. (2007). Tissue Engineering.

[34] Franke J., Abs V., Zizzadoro C., Abraham G. (2014). Comparative study of the effects of fetal bovine serum versus horse serum on growth and differentiation of primary equine bronchial fibroblasts. BMC Vet. Res..

[35] Naito H., Melnychenko I., Didié M., Schneiderbanger K., Schubert P., Rosenkranz S., Eschenhagen T., Zimmermann W.-H. (2006). Optimizing Engineered Heart Tissue for Therapeutic Applications as Surrogate Heart Muscle. Circulation.

[36] Ribeiro M.C., Tertoolen L.G., Guadix J.A., Bellin M., Kosmidis G., D’Aniello C., Monshouwer-Kloots J., Goumans M.-J., Wang Y.-L., Feinberg A.W. (2015). Functional maturation of human pluripotent stem cell derived cardiomyocytes in vitro—Correlation between contraction force and electrophysiology. Biomaterials.

[37] Bellin M., Casini S., Davis R., D’Aniello C., Haas J., Oostwaard D.W.-V., Tertoolen L.G.J., Jung C.B., Elliott D., Welling A. (2013). Isogenic human pluripotent stem cell pairs reveal the role of a KCNH2 mutation in long-QT syndrome. EMBO J..

[38] Eder A., Vollert I., Hansen A., Eschenhagen T. (2016). Human engineered heart tissue as a model system for drug testing. Adv. Drug Deliv. Rev..

[39] Maier L.S., Schwan C., Schillinger W., Minami K., Schütt U., Pieske B. (2000). Gingerol, isoproterenol and ouabain normalize impaired post-rest behavior but not force-frequency relation in failing human myocardium. Cardiovasc. Res..

[40] Nguyen N., Nguyen W., Nguyenton B., Ratchada P., Page G., Miller P.E., Ghetti A., Abi-Gerges N. (2017). Adult Human Primary Cardiomyocyte-Based Model for the Simultaneous Prediction of Drug-Induced Inotropic and Pro-arrhythmia Risk. Front. Physiol..

[41] Birket M., Casini S., Kosmidis G., Elliott D., Gerencser A.A., Baartscheer A., Schumacher C., Mastroberardino P.G., Elefanty A., Stanley E.G. (2013). PGC-1α and Reactive Oxygen Species Regulate Human Embryonic Stem Cell-Derived Cardiomyocyte Function. Stem Cell Rep..

[42] Polonchuk L., Surija L., Lee M.H., Sharma P., Ming C.L.C., Richter F., Ben-Sefer E., Rad M.A., Sarmast H.M.S., Al Shamery W. (2021). Towards engineering heart tissues from bioprinted cardiac spheroids. Biofabrication.

[43] Stein J.M., Mummery C.L., Bellin M. (2021). Engineered models of the human heart: Directions and challenges. Stem Cell Rep..

[44] Giacomelli E., Bellin M., Sala L., van Meer B.J., Tertoolen L.G.J., Orlova V.V., Mummery C.L. (2017). Three-dimensional cardiac microtissues composed of cardiomyocytes and endothelial cells co-differentiated from human pluripotent stem cells. Development.

