# Supplementary material for: A New Versatile Platform for Assessment of Improved Cardiac Performance in Human-Engineered Heart Tissues
Source: J Pers Med. 2022 Feb 4;12(2):214. doi: 10.3390/jpm12020214 (PMC8877418; doi:10.3390/jpm12020214)
Supplement: Supplementary file 1 [file jpm-12-00214-s001.zip › jpm-1522264-supplementary.pdf]

Supplementary Materials

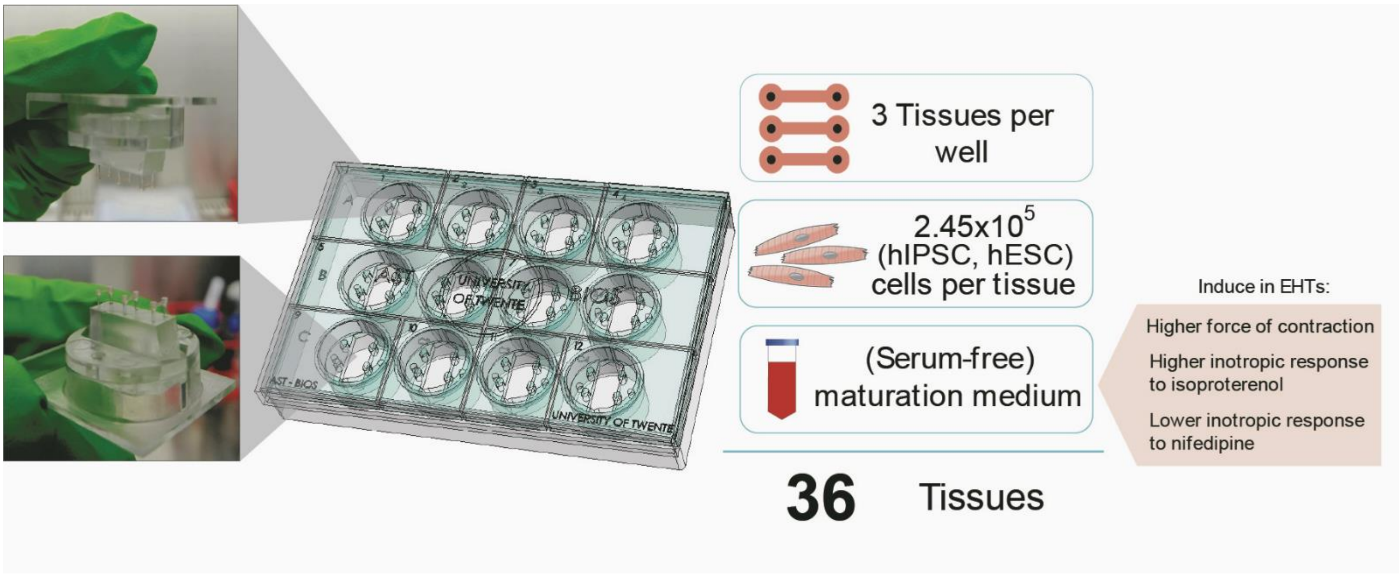

Figure S1. Schematic of the main advantages of the versatile platform for assessment of human engineered heart tissues

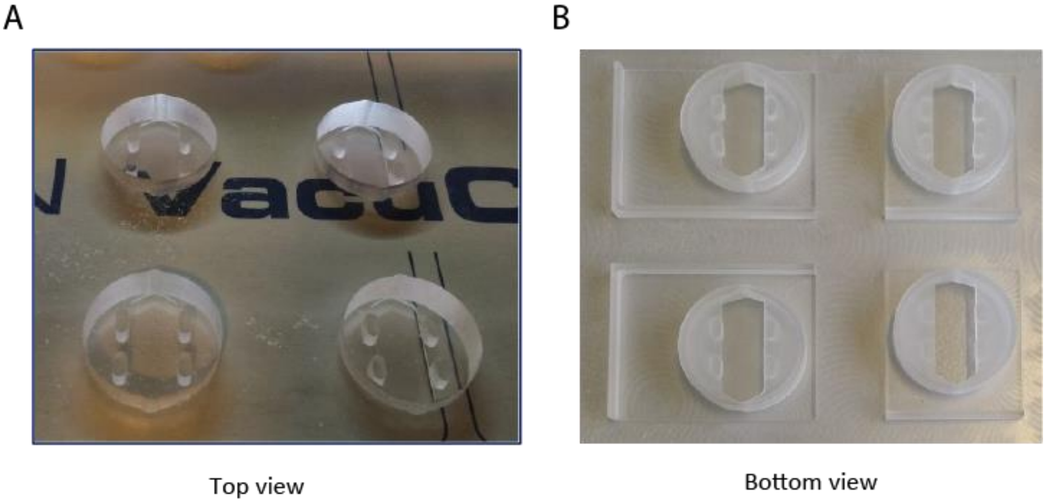

Figure S2. Holders fabrication from PMMA by a milling machine. (A) Top part of the set of 4 holders from PMMA (B) Bottom part of the set of 4 holders from PMMA.

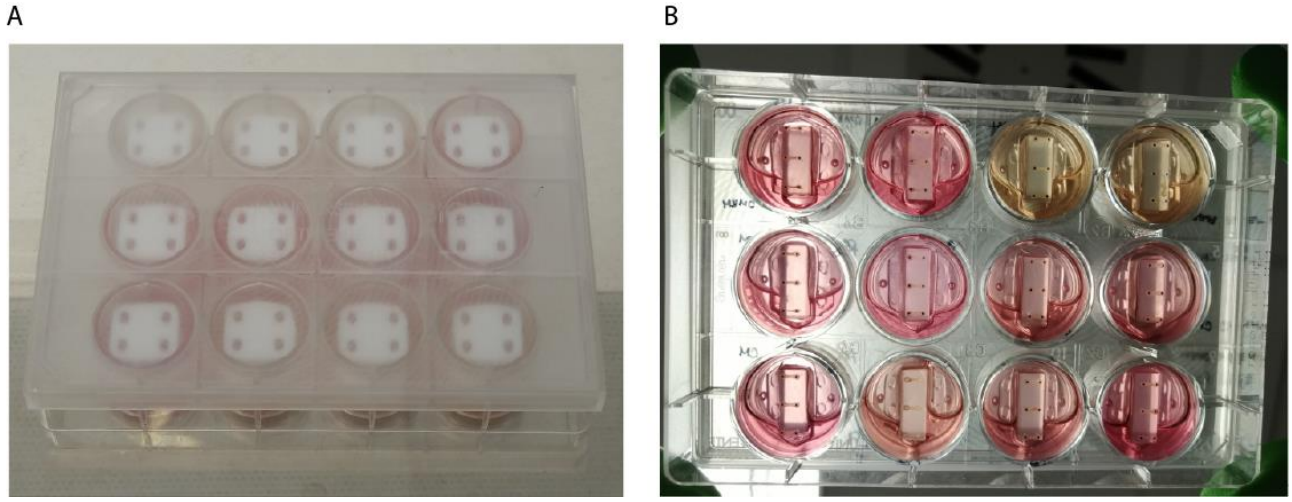

Figure S3. Bioengineered platform (A) Top view of the platform with 12 spacers. (B) Bottom view of the platform with 36 EHTs in different media.

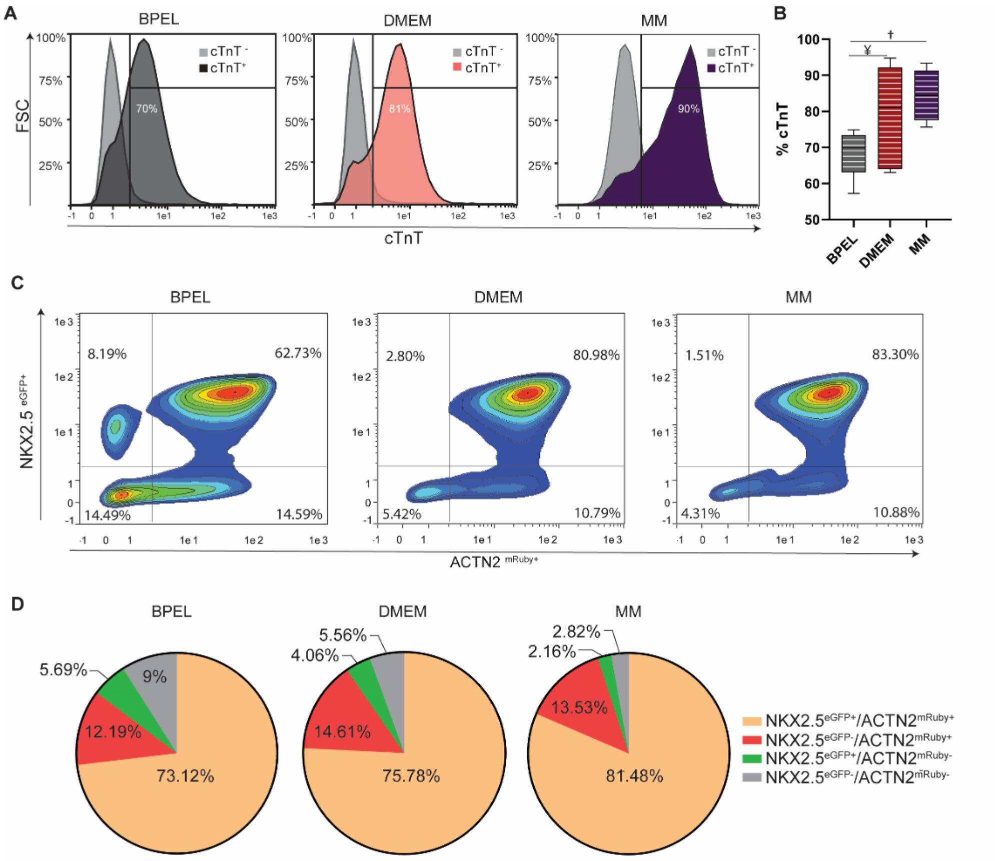

Figure S4. Cardiomyocyte differentiation. Flow cytometry (FC) characterization of differentiated CMs at D17 in different media. (A) Representative hiPSC-CM histograms of cardiac troponin T positive cells (cTnT). (B) Average hiPSC-CM differentiation efficiency with cardiac troponin T percentages (% cTnT). Data shown as One-way ANOVA plus Tukey's test. Values expressed as means ± SEM. ¥ = $p < 0.05$; † = $p < 0.01$.(C) Representative hESC-CM density plots of ACTN2-(ACTN2mRuby+) and NKX2.5 (NKX2.5eGFP+) positive cells. (D) Average hESC-cardiomyocyte differentiation efficiency pie charts.

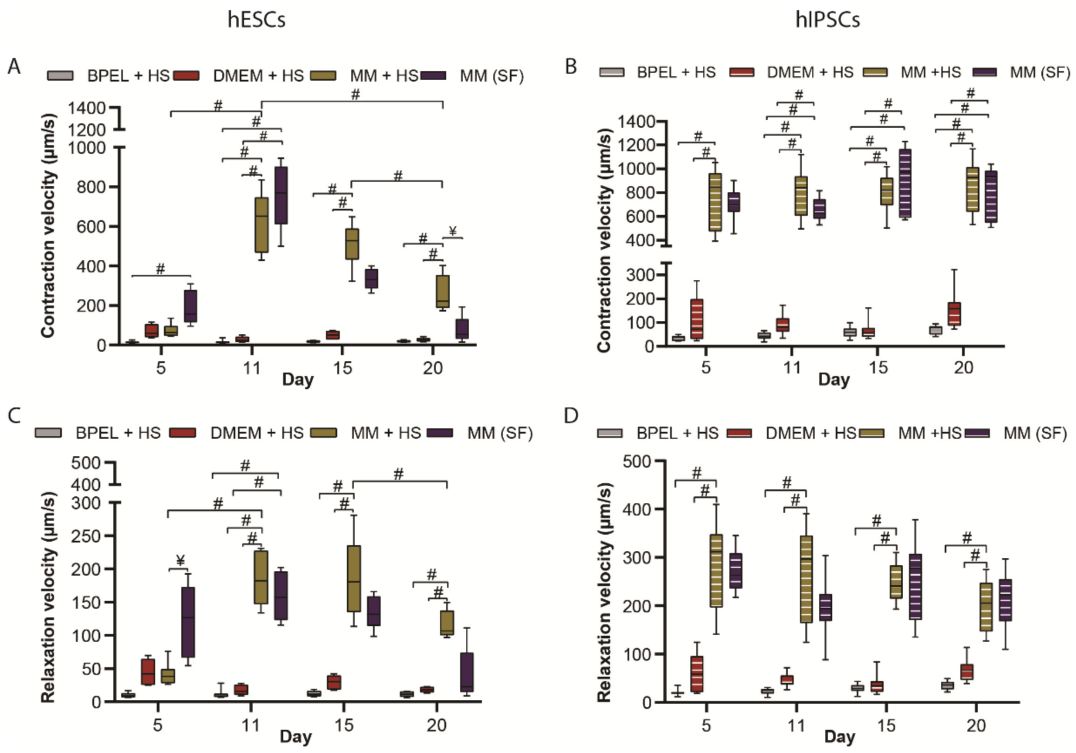

Figure S5. Contraction & Relaxation velocity in different media. (A-B) Contraction velocity in hESCs (A) and hiPSCs (B) at day 5, 11, 15 and 20. (C-D) Relaxation velocity in hESCs (C) and hiPSCs (D) at day 5, 11, 15 and 20. Data shown as means, maxima and minima; Two-way ANOVA plus Tukey's test for comparisons among media; † = p <0.01; ‡ = p <0.001; # = p <0.0001. HS = Horse serum; SF = serum free.

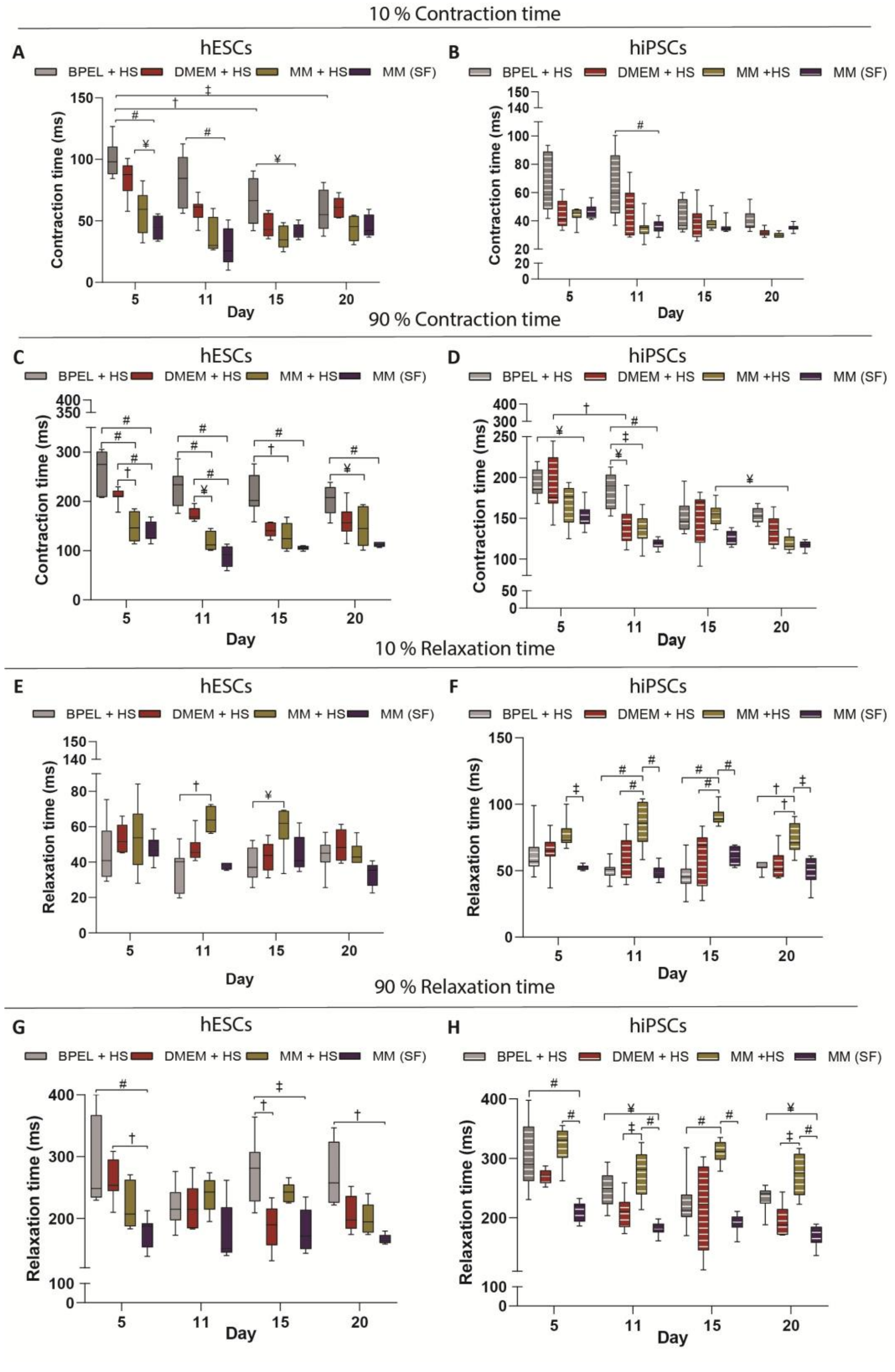

Figure S6. Contraction & Relaxation time in different media. (A-B) Time to reach the 10 % of contraction in hESCs (A) and hiPSC (B). (C-D) Time to reach the 90 % of contraction in hESCs (C) and hiPSC (D). (E-F) Time to reach the 10 % of relaxation in hESCs (E) and hiPSC (F) (G-H) Time to reach the 90 % of relaxation in hESCs (G) and hiPSC (H). Data shown as means, maxima and minima; Two-way ANOVA plus Tukey's test; ¥ = p <0.05; † = p <0.01; ‡ = p <0.001; # = p <0.0001. HS = Horse serum; SF = serum free.

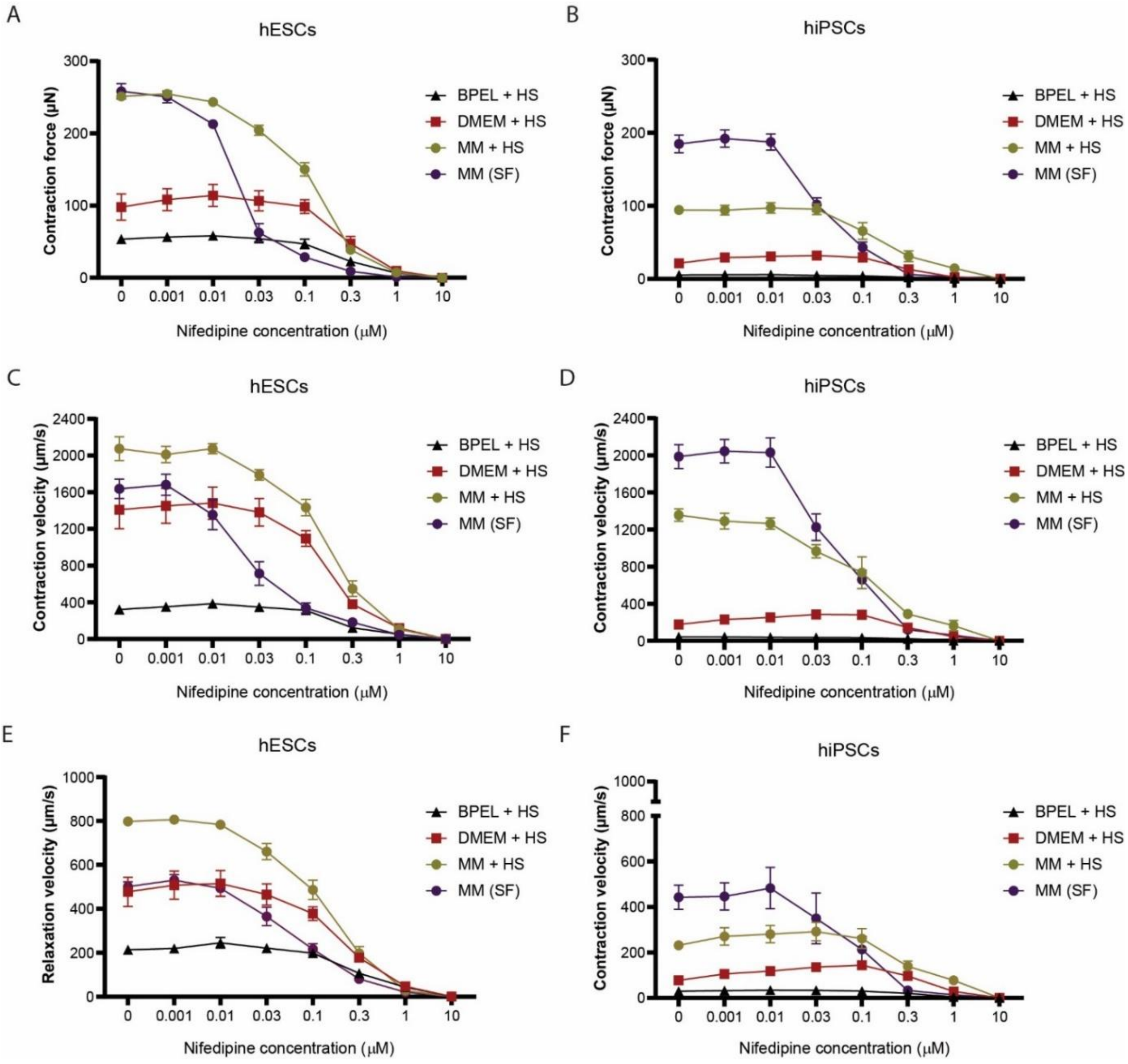

Figure S7. Response to negative inotropic agents in hESC- and hiPSC-EHTs. A-B. Contraction force of hESCs (A) and hiPSCs (B) EHTs in different media in response to nifedipine (0-10 μM). C-F. Contraction and relaxation velocity in different media in response to nifedipine (0-10 μM). C-D. Contraction velocity in hESCs (C) and hiPSCs (D). (E-F) Relaxation velocity in hESCs (E) and hiPSCs (F). A-F. Values are expressed as means ± SEM. HS = Horse serum; SF = serum free.

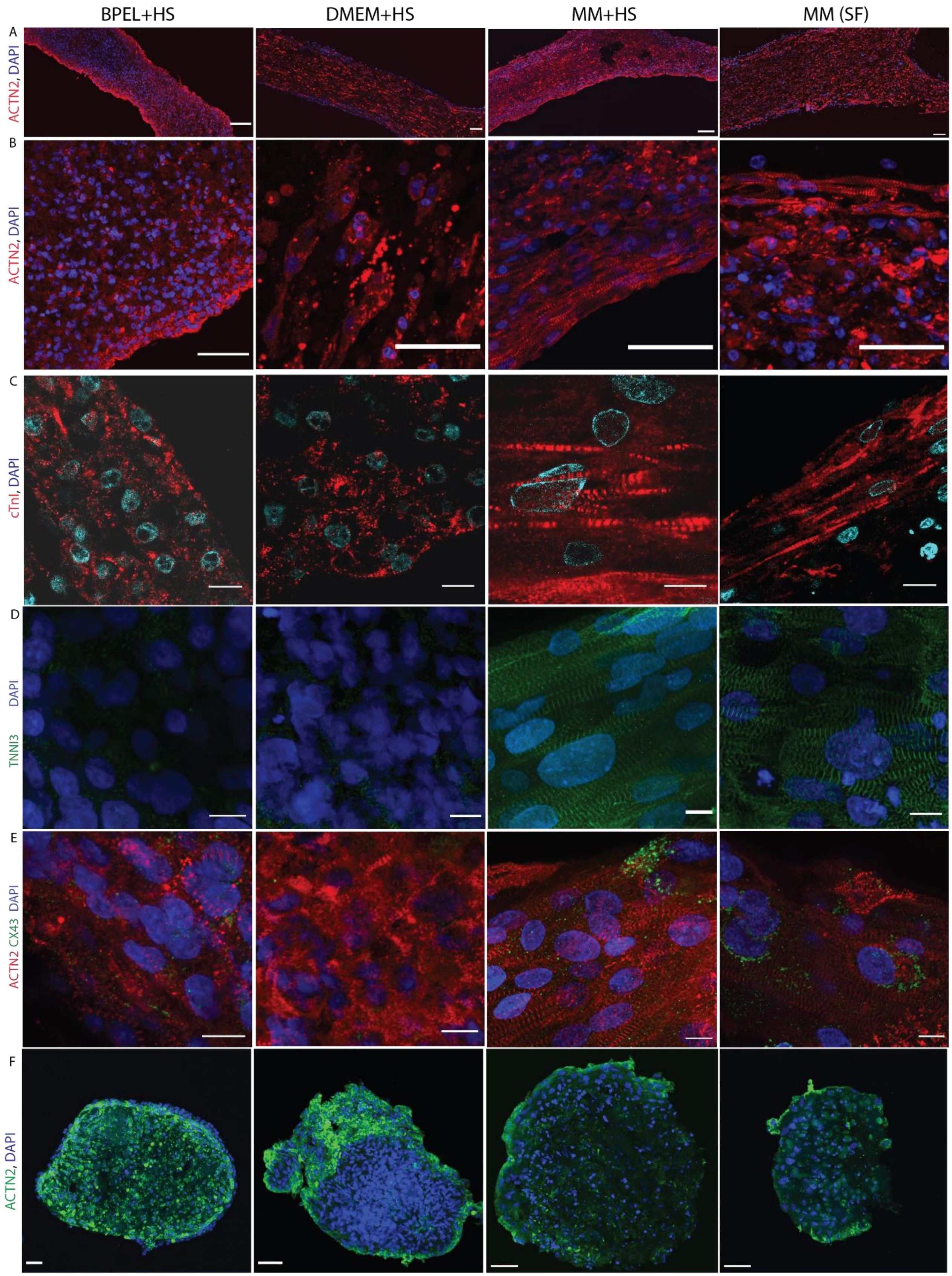

Figure S8. Morphological analysis of EHTs. A-B. Representative tissue cryosections immunostained for α-actinin (ACTN2, red) and DAPI (nuclei, blue). C. Representative paraffin sections of EHTs immunostained for cardiac troponin-I (cTnI, red) counterstained with DAPI (nuclei, blue). D-E. Confocal images of whole mount tissue immunostaining for cardiac troponin I (TNNI3, green)(D) or α-actinin (ACTN2, red) and Connexin 43 (CX43, green)(E), counterstained with DAPI (nuclei, blue). F. Representative transversal cryosections immunostained for α-actinin (ACTN2, green) counterstained with DAPI (nuclei, blue). (A) Scale bars, 100 μm, (B,F) Scale bars, 50 μm, (C, D, E) Scale bars, 10 μm. HS = Horse serum; SF = serum free.

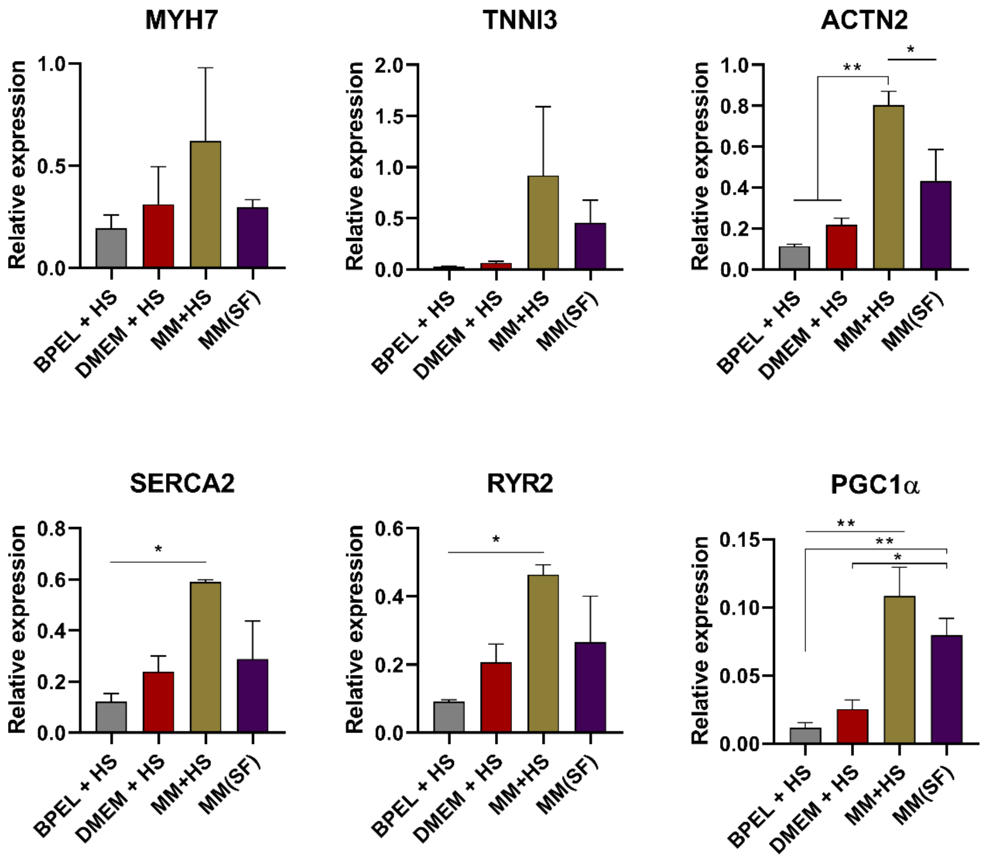

Figure S9. Relative gene expression of cardiac genes for hESC-CM BPEL+HS, DMEM+HS, MM+HS or MM(SF) at day 21. Expression levels were normalized to RPLP0 expression. Values are expressed as means ± SEM. * = $p < 0.05$; ** = $p < 0.01$. HS = Horse serum; SF = serum free.

Supplementary Table S1. Primer sequences for RT-qPCR

Primer	Forward	Reverse
RPLP0	CACCATTGAAATCCTGAGTGATGT	TGACCAGCCCAAAGGAGAAG
MYH7	CGCACCTTCTTCTCTTGCTC	GAGGACAAGGTCAACACCCT
TNNI3	CAGTAGGCAGGAAGGCTCAG	CCTCAAGCAGGTGAAGAAGG
ACTN2	CTGCTGCTTTGGTGTCAGAG	TTCCTATGGGGTCATCCTTG
SERCA2	ACCCACATTCGAGTTGGAAG	CCAACGAAGGTCAGATTGGT
RYR2	AAGCCTCCGTCTGAAACA	CCACCCAGACATTAGCAGGT
PGC1α	AACACTTACAAGCCAAACCA	GGGTTCAATAGTCTTGTTCTC
